# Artesunate Suppresses the Proliferation and Development of Estrogen Receptor-α-Positive Endometrial Cancer in HAND2-Dependent Pathway

**DOI:** 10.3389/fcell.2020.606969

**Published:** 2021-01-12

**Authors:** Xianghua Yin, Yan Liu, Jiarui Qin, Yixuan Wu, Jiayan Huang, Qi Zhao, Tingting Dang, Yacui Tian, Ping Yu, Xiyue Huang

**Affiliations:** ^1^Department of Obstetrics and Gynecology, Clinical Medical College of Yangzhou University, Yangzhou, China; ^2^Northern Jiangsu People’s Hospital, Yangzhou, China; ^3^Department of Urology, Medical College, Yangzhou University, Yangzhou, China; ^4^Department of Gynaecology and Obstetrics, Subei People’s Hospital of Jiangsu Province, Yangzhou, China

**Keywords:** endometrial cancer, artesunate, ER-α-positive, HAND2, artesunate (ART)

## Abstract

Endometrial cancer (EC) is a common leading cause of cancer-related death in women, which is associated with the increased level of estrogen in the body. Artesunate (ART), an active compound derived from *Artemisia annua* L., exerted antitumor properties in several cancer types. However, the role of artesunate and the molecular basis on EC remains unclear. Here, we aimed to explore the effects and mechanisms of artesunate. Our results identified that estrogen receptor-α (ER-α) was a key factor for the type I EC (ER-α-positive), which might suppress the downstream LKB1/AMPK/mTOR pathway. Besides, we found ART significantly inhibited tumor proliferation in a dose-dependent manner. Mechanistic studies identified that ART led to tumor cell apoptosis and cell cycle arrest by downregulating the ER-α expression and activating the LKB1/AMPK/mTOR pathway. In addition, we found ART could increase the expression of heart and neural crest derivatives expressed 2 (HAND2) in the ER-α-positive EC cells, which could interact with ER-α. Through the gain-and loss-function experiments, we showed that over expression of HAND2 repressed the proliferation and migration of ER-α-positive EC cells via inhibition of ER-α expression. HAND2 knockdown increased ER-α expression and alleviated the antitumor effect of ART *in vitro* and *in vivo*. Overall, our study first showed that ART could be an effective antitumor agent through modulating ER-α-mediated LKB1/AMPK/mTOR pathway in the HAND2 dependent manner. Our findings provide an effective therapeutic agent for ER-α-positive EC treatment.

## Introduction

Endometrial cancer (EC) is a common cause of cancer-related death worldwide, affecting about 21% of women (Sorosky, 2012; Brooks et al., 2019). Multiple risk factors contributed to EC formation, including reproductive factors, imbalanced hormone, age, and obesity (Amant et al., 2005). Estrogen is an essential hormone that exerts multiple physiological functions, such as tissue growth, modulation of brain function, or bone density (Liang and Shang, 2013). The action of estrogen relies on the estrogen receptor (ER) including ER-α and ER-β subtypes (Shah and Rowan, 2005). Researchers identified that sustained stimulation with exogenous estrogen is a crucial risk factor in accelerating the progression of ER-related cancers. Indeed, almost 80% of the EC are categorized into type I EC [endometrioid-type endometrial cancer (EEC)] which is estrogen-positive and present at the early stage with an excellent prognosis (Amant et al., 2005). Positivity for ER-α is pervasive in EEC, and serves as the prognostic marker (Jongen et al., 2009). Studies revealed that ER-α rapidly modulated the AMP-activated protein kinase (AMPK) signaling pathway, which involved in the modulation of cell proliferation, differentiation, and death. Treatment with the specific ER-α agonist has been identified to interact with the upstream liver kinase B 1 (LKB1) kinase complex, which is a tumor suppressor gene and activate APMK to accelerate tumorigenesis (Faubert et al., 2013; Lipovka et al., 2015). In many types of cancer, including EC, AMPK was inactivated due to the high glucose metabolic conditions (Han et al., 2015). The role of the ER-α-mediated LKB1/AMPK pathway in EC, however, is still unexplored.

From the clinical statement of EEC, endocrine therapy, and chemotherapy seem to decrease the incidence and mortality of EEC. But it has not been widely used due to its severe side effects and drug resistance (Gao et al., 2014). Therefore, it is still essential to further investigate new therapeutic strategies for EC. Currently, researchers increasingly focused on traditional medicine because of the low side effects. Artesunate (ART), a natural product isolated from *Artemisia annua* L., possesses many different bioactivities, including antiviral, anti-fibrosis, anti-inflammatory, and immunoregulation activities (Efferth et al., 2008; Meng et al., 2018; Kong et al., 2019; Li et al., 2019). ART exerted anti-inflammatory effects in leishmaniasis-induced neuroinflammation and in traumatic brain injury to play neuroprotective effect (Gugliandolo et al., 2018, 2020). ART also inhibited cell proliferation and migration of cancer cells by regulating the oxidative stress response, DNA damage and repair, inducing various cell death forms, and controlling several tumor-associated pathways (Efferth et al., 2003; Berdelle et al., 2011; Zhang et al., 2018; Zheng and Pan, 2018). It is worth noting that ART could activate AMPK and suppress mTOR signaling in the model of oral tongue squamous cell carcinoma (Xiao et al., 2020). Nevertheless, the cytotoxic effect of ART on EC cells and the molecular basis remain unclear.

Accordingly, we aimed to elucidate whether the ER-α-mediated LKB1/AMPK pathway played an effective role in the EC progression and to reveal whether ART could be used as a novel agent for EC treatment. In this study, we examined the effect of ART in two ER-α-positive EC cell lines, and found that ART suppressed cell proliferation and induced cell apoptosis. The administration of ART decreased the expression of ER-α and activated the downstream pathway. Moreover, based on the *in vitro* and *in vivo*, we showed that ART upregulated the heart and neural crest derivatives expressed 2 (HAND2) to inhibit ER-α expression. Taken together, we explored that ART is an effective therapeutic agent for ER-α-positive EC treatment.

## Materials and Methods

### Clinical Sample

The EC tumor tissues (*n* = 100) and normal endometrial tissue samples (*n* = 100) were obtained from the patients who were diagnosed with EC. The age of the EC patients was range from 33 to 70 years old, with the average age was 51.60 ± 7.83. This experiment was approved by the Ethics Committee of Northern Jiangsu People’s Hospital (2017KY-052), and written informed consent was obtained from all patients.

### Quantitative Reverse Transcription-Polymerase Chain Reaction (qRT-PCR)

The procedure of qRT-PCR was performed to determine the mRNA level (Wang et al., 2019). According to the manufacturer’s instructions, total RNAs were extracted from the cells and tissues using Trizol reagent (Thermo, United States). Next, 1 μg of total RNA was reverse-transcribed into cDNA using TaqMan Reverse Transcription Kit (Applied Biosystems, United States). Then, qRT-PCR was performed to detect the mRNA level by SYBR green (Toyobo, Japan). The data were measured using the 2^–ΔΔCT^ method and normalized by GAPDH. The primers used in this study were presented as follows: ER-α: forward 5′-TTATCAGACCAAGGCCTCACA C-3′, reverse 5′-TCCCACAGCCTTGTCTTGGT-3′; LKB1: for- ward 5′-CAGAAGAGGAGGCCAGTCAC-3′, reverse 5′-AAA GGCCACATGGCAACCAC-3′; AMPK: forward 5′-CGAGAAG CAGAAACACGACG-3′, reverse 5′-ATGAAGAGGTGCGGAA AGCG-3′; HAND2: forward 5′-CAAAATCAAGACCC TGCGCC-3′, reverse 5′-ACAACCTGGTGCAAACAACC-3′; GAPDH: forward 5′-AAGGGCCCTGACAACTCTTTT-3′, reverse 5′CTGGTGGTCCAGGGGTCTTA-3′; mTOR: forward 5′-TATTTGAACAGTCCCCGCCC-3′, reverse 5′-CGTGGGT CTGGACATTACGC-3′.

### Immunohistochemical Analysis

Immunohistochemical analysis was performed as previously described (Dominguez et al., 2018). The tumor or normal tissues were collected and perfused with 10% formaldehyde solution for 24 h at 4°C, followed by embedment in paraffin. The paraffin blocks were then cut into sections with a thickness of 5 μm. The sections were deparaffinized, rehydrated, heated, and blocked the non-specific antigen with 5% BSA for 1 h. Then the sections were incubated with primary antibodies for HAND2 (1:500, Abcam), ER-α (1:300, Abcam), LKB1 (1:500, Abcam), AMPK (1:500, Abcam), and mTOR (1:500, Abcam) at 37°C overnight, followed by visualized using the DAKO REAL EnVision Inspection System (DAKO, Denmark). All images were observed under a light microscope (Nikon, Japan).

### Western Blot

The protein expression of cells or tissues was detected by using western blot as previously described (Jing W. et al., 2019). Cells or tissue samples were collected and homogenized using RIPA buffer. Equal total proteins (30 μg/lane) were separated by SDS-PAGE (Bio-Rad, United States), and transferred onto the PVDF membrane. Next, the membranes were blocked by 5% BSA for 2 h at room temperature, followed by incubation with primary antibodies against ER-α (1:1000, Abcam, United States), LKB1 (1:1000, Abcam, United States), p-LKB1 (1:1000, Abcam, United States), AMPK (1:1000, Abcam, United States), p-AMPK (1:1000, Abcam, United States), mTOR (1:1000, Abcam, United States), p-mTOR (1:1000, Abcam, United States), HAND2 (1:1000, Abcam, United States), Lamin B1 (1:5000, Abcam, United States), and GAPDH (1:5000, Abcam, United States) at 4°C overnight. After washing with 1 × PBS for three times, the membranes were incubated with HRP-conjugated secondary antibodies (1:5000, Abcam, United States) for 2 h at room temperature. The bands were then developed using enhanced chemiluminescence chromogenic substrate (GE Healthcare, United Kingdom) and analyzed by the Image J software. Lamin B1 or GAPDH was used as a control in this study.

### Cell Culture and Transfection

The Ishikawa and HEC-1-A cell lines were obtained from ATCC (Manassas, United States) and maintained with DMEM culture medium (Gibco, United States) added 10% fetal bovine serum (Gibco, United States), 1% penicillin and streptomycin (Thermo, United States). For the *in vitro* transfection, cells were seeded onto the 3-cm dishes at the density of 10^6^ cells per well, and incubated with 5 μg of HAND2 overexpressed or knockdown vectors (HAND2-OE, HAND2-KD, GenePharma, China) using lipofectamine 2000 (GenePharma, China) following the manufacturer’s instructions. After 48 h, the cells were used for further studies.

### Cell Treatment

The dose and conditions of multiple agents were as follows: ART (Sigma, United States) was dissolved in PBS with a final working concentration of 0–30 μg/mL for 0–6 days; Fulvestrant (MedChemExpress, United States) was dissolved in DMSO and treated cells at the dose of 250 nM; 10 nM of estrogen (Sigma, United States) was diluted using PBS. Pim1/AKK1-IN-1 (380 nM in DMSO, MedChemExpress, United States) was performed to treat cells. After 4 days, the cells were collected for further study.

### Cell Counting Kit-8 (CCK-8) Assay and Colony Formation Assay

Cell proliferation was measured by cell counting kit (CCK-8) assay and colony formation assay (Zhang et al., 2019). Cells were seeded on 96-well plates (5000 cells/well) and subjected to different treatments. Next, the cells were then treated with 10 μL of CCK-8 solution (Beyotime, China) at 37°C for 2 h. The optical density (OD value) of each well was measured by a microplate reader at 450 nm. For the colony formation assay, cells were plated at the density of 5000 cells per well. After different treatments, cells were cultured with a fresh medium for 10 days. The colonies were fixed with methanol, dyed by hematoxylin, and observed under the light microscope (Nikon, Japan).

### Cell Cycle and Apoptosis Analyses

The cell cycle and apoptosis were analyzed by using flow cytometry (Mohammed et al., 2018). The effect of ART on cell cycle and apoptosis was assessed by flow cytometry using the Annexin V Apoptosis Detection kit (BD Biosciences, United States) and the Cell Cycle kit (BD Biosciences, United States) following the manufacturer’s protocol. In brief, for cell cycle analysis, the cells were collected after different treatments, followed by fixation with 70% ethanol at 4°C overnight and staining with propidium iodide (PI) solution (0.1 μg/μL) in the presence of Ribonuclease A (Takara) at 37°C for 30 min in the dark. For the apoptosis analysis, cells were stained with Annexin V-FITC and PI (BD Biosciences) for 15 min in the dark at room temperature. Then the cells were evaluated by flow cytometry (FACScan; BD Biosciences). The data were analyzed using FlowJo v10 software (Tree Star, United States).

### Migration

The migration ability of cells was assessed using the *trans*-well system (Costar, United States) as previously described with some modification (Zhao et al., 2017). Briefly, after different treatments, 1 × 10^5^ cells were seeded onto the upper chamber and incubated with the serum-free medium. The medium containing 10% FBS was added onto the lower chambers. After 48 h, the migrated cells were fixed with 4% paraformaldehyde, stained with 10% crystal violet at room temperature, and observed under a light microscope in five random fields (200×).

### Animal and Treatment

Female BALB/c nude mice (4 weeks, 10–15 g) were obtained from the Animal Center of Nanjing University (Nanjing, China). After different transfection, the Ishikawa cells (10^6^ cells in 100 μL PBS) were injected into mice subcutaneously to form tumors as previously described (Yang et al., 2020). The mice were then divided into four groups: control group, HAND2-KD group, control + ART group, and HAND2-KD + ART group (*n* = 10 of each group). ART-treated mice were received ART daily (30 mg/kg, in 200 μL PBS) via intramuscular injection. The control mice were treated by an equal volume of PBS. Tumor size was recorded every week and measured according to the formula tumor volume = (length × width^2^)/2. After 5 weeks, the tumor tissues were removed for weighting and further studies when the mice were sacrificed. All animal experiments were approved by the Ethics Committee of Northern Jiangsu People’s Hospital (SCXK(Su)2017-0007).

### Statistical Analysis

Data were analyzed using GraphPad Prism 8.0 software and are presented as the mean ± SD. Two-tailed Student’s *t*-test was used to compare the difference between the two groups. One-way ANOVA followed by Tukey’s *post hoc* test was performed to compare the difference among multiple groups. *P* < 0.05 was considered statistically significant.

## Results

### ER-α Suppressed the LKB1/AMPK Pathway in the ER-α-Positive EC

**FIGURE 1:**
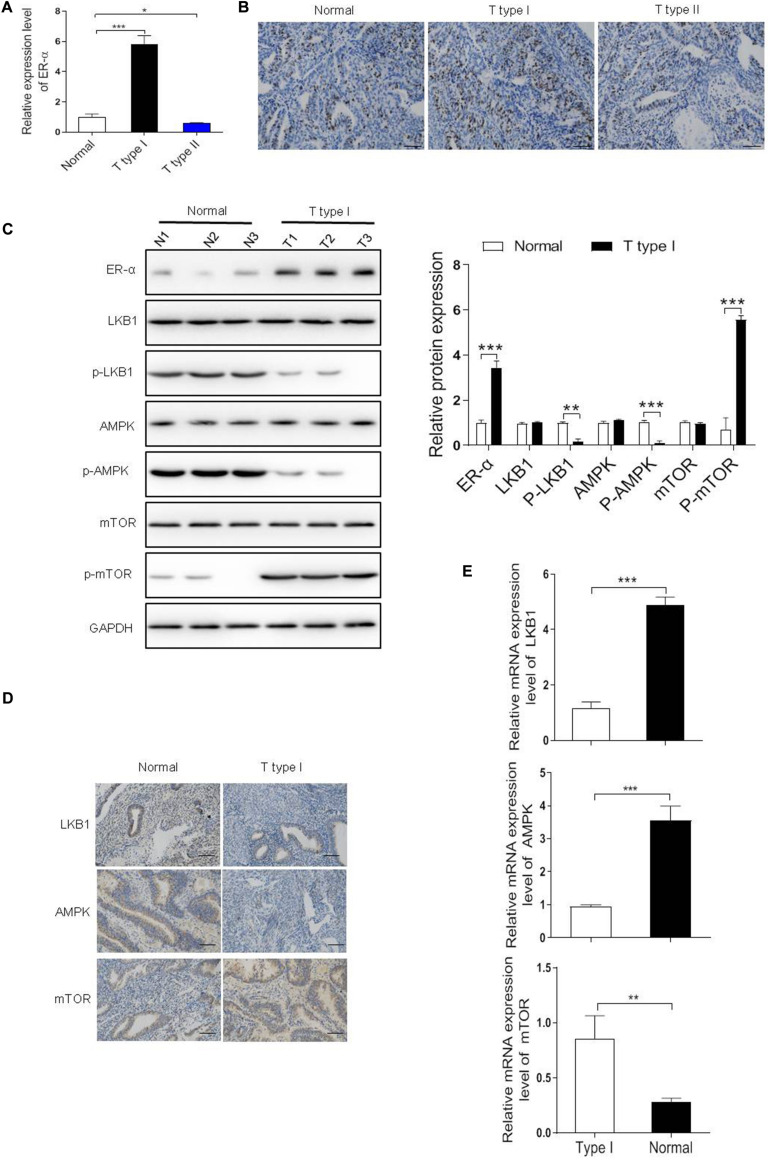
Estrogen receptor-α (ER-α) suppressed the LKB1/AMPK pathway in the ER-α-positive EC. **(A,B)** The expression level of ER-α in different subtypes of EC clinical samples and normal endometrial tissues were assessed by qRT-PCR and IHC assays. Bar = 100 μm. **(C)** Representative blot and analyses were performed for the protein levels of ER-α, LKB1, p-LKB1, AMPK, p-AMPK, mTOR, p-mTOR in type I EC tumor samples and normal endometrial tissues (*n* = 3). **(D,E)** The expression level of LKB1, AMPK, mTOR in type I EC tumor samples and normal endometrial tissues were assessed by qRT-PCR and IHC assays. Bar = 100 μm. The results of quantification were showed as the mean ± SD, *p < 0.05, **p < 0.01, ***p < 0.001 vs. the normal group.

Firstly, we detected the expression level of ER-α in our clinical samples (*n* = 100) and found that compared with the normal tissue samples (*n* = 100), a six-fold upregulation of ER-α expression was detected in the type I EC samples (*n* = 72), while a 50% reduction was observed in the type II EC samples (*n* = 28), suggesting that more than 72% EC was estrogen-sensitive (Figure 1A). A similar trend was observed in the IHC images of these tissue samples, with the type I EC showing more positive expression of ER-α (Figure 1B).

In ERα-positive breast cancer cells, LKB1 binds to ERα and in doing so makes its cytosolic pool not adequate to activate AMPK signaling, thus maintaining mTOR signaling and sustaining cell growth (Ando et al., 2020). To investigate the biological effect of ER-α on tumorigenesis in ER-α-positive EC, mRNA and protein levels of genes in the LKB1/AMPK/mTOR pathway were determined. As shown in Figure 1C,E, the mRNA and protein levels of p-LKB1 and p-AMPK were both markedly decreased whereas the p-mTOR expression was dramatically increased in the type I EC samples compared with the normal group, suggesting that the activation of LKB1/AMPK/mTOR pathway might be associated with the clinical status of ER-α in EC. Besides, as shown in Figure 1D, a higher level of LKB1, AMPK, and mTOR was observed in the normal endometrial tissues than the type I tumor samples.

### ART Suppressed Cell Proliferation and Cell Death in ER-α-Positive EC Cells

**FIGURE 2:**
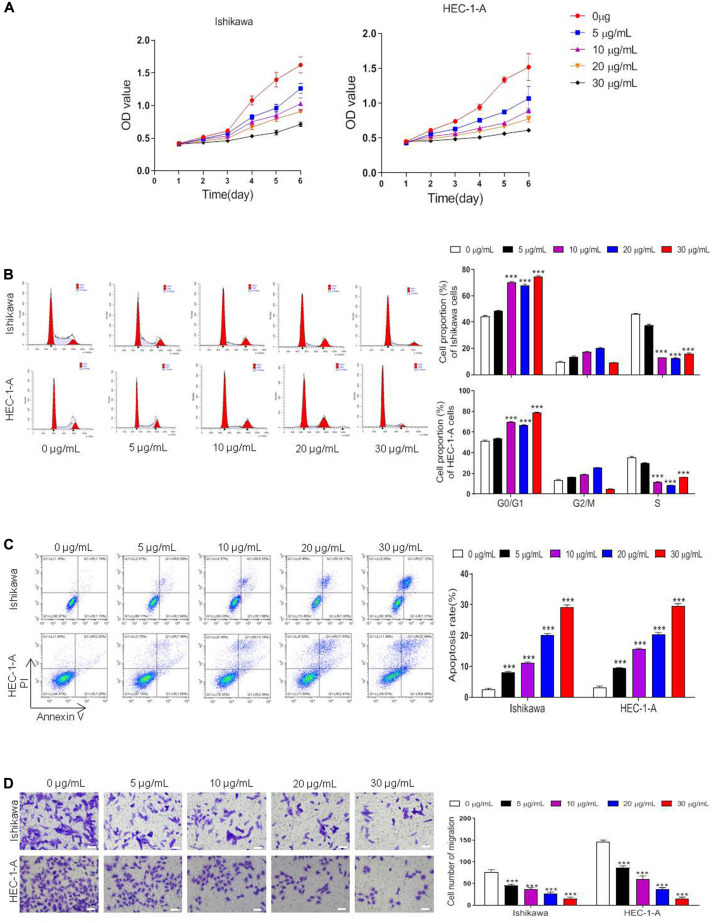
Artesunate (ART) inhibited the proliferation of Ishikawa and HEC-1-A cells. Cells were treated with ART at different concentrations (0–30 μg/mL) for 1–6 days. **(A)** Cell viability was measured with the CCK-8 assay. **(B–D)** The cell cycle analysis, apoptosis, and migration assays were performed in these cells after different treatments. Bar = 20 μm. The data were showed as the mean ± SD, ***p < 0.001 vs. the 0 μg/mL of ART-treated group.

To investigate whether ART suppressed cell proliferation in ER-α-positive EC cells, we treated Ishikawa and HEC-1-A cells with 0, 5, 10, 20, 30 μg/mL of ART for 1–6 days and performed CCK-8 cytotoxicity assay. The results showed that the cell viability of these cells was both inhibited dose-dependently (Figure 2A). We also checked whether ART induced cell cycle arrest using flow cytometry analysis. These cells were stimulated with 0–30 μg/mL of ART for 4 days, and the results showed that ART induced cell arrest in the G0/G1 phase in the dose-dependent manner (Figure 2B). Besides, as shown in Figure 2C, ART administration led to apoptotic cell death concentration-dependently in both cell lines. Next, the cell migration ability of such cells was assessed using the *trans*-well assay. The result showed a markedly decrease in the migrated cells in Ishikawa and HEC-1-A cells after ART treatments (Figure 2D).

### ART Regulated Cell Proliferation Through the ER-α Related LKB1/AMPK/mTOR Pathway

**FIGURE 3:**
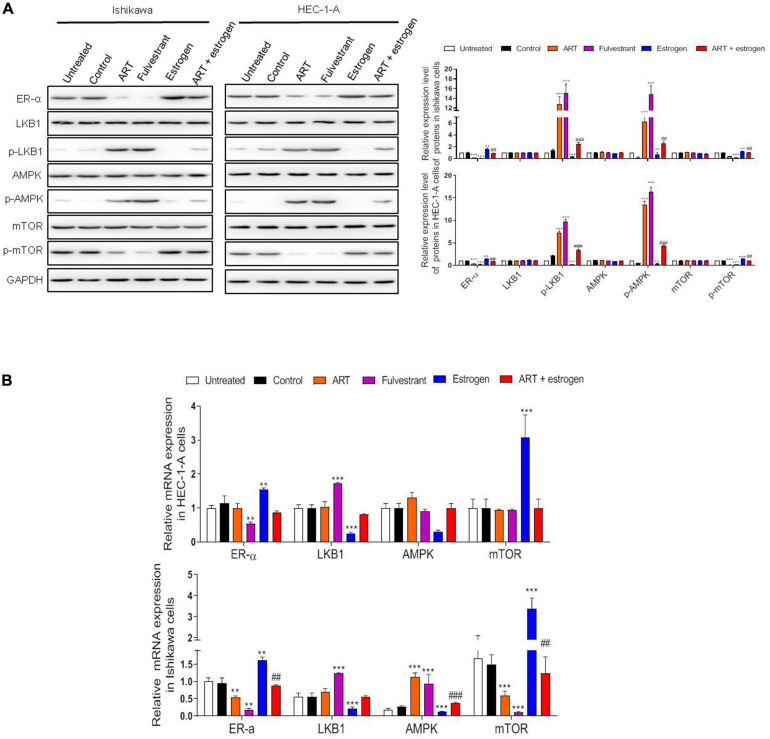
Artesunate (ART) inhibited the proliferation of Ishikawa and HEC-1-A cells via regulating the ER-α related LKB1/AMPK/mTOR pathway. Ishikawa and HEC-1-A cells were treated with PBS, ART (30 μg/mL), fulvestrant (250 nM), estrogen (10 nM) or ART + estrogen for 4 days. **(A)** Representative blot and analysis were performed for the protein levels of ER-α, LKB1, p-LKB1, AMPK, p-AMPK, mTOR, p-mTOR in these cells. **(B)** The mRNA levels of these genes were performed by qRT-PCR analysis. The data were showed as the mean ± SD, **p < 0.01, ***p < 0.001 vs. the control group; ##*p* < 0.01, ###*p* < 0.001 vs. the ART-treated group.

As shown in Figure 3A, the treatment of Ishikawa and HEC-1-A cells with ART (30 μg/mL) significantly decreased ER-α protein expression, which was consistent with the result of 250 nM fulvestrant (the antagonist of estrogen receptor) treatment. As shown, exposure to 10 nM estrogen (the agonist of estrogen receptor) dramatically upregulated the expression of ER-α, while this effect was reversed when combination treated with ART together. Further, we tested the influence of ART on the ER-α related LKB1/AMPK/mTOR pathway. The levels of p-LKB1 and p-AMPK increased while the expression of p-mTOR decreased in these cells after ART or fulvestrant treatment. However, when the cells were stimulated by estrogen, the expression of LKB1 and AMPK was inactivated whereas the level of p-mTOR was increased compared with the control group. ART could also abrogate the effect of estrogen on this pathway. A similar trend was also determined at the mRNA level of such genes (Figure 3B). These data suggest that ART can relieve proliferation through ER-α related LKB1/AMPK/mTOR pathway *in vitro*.

### ART Increased the Expression of HAND2

**FIGURE 4:**
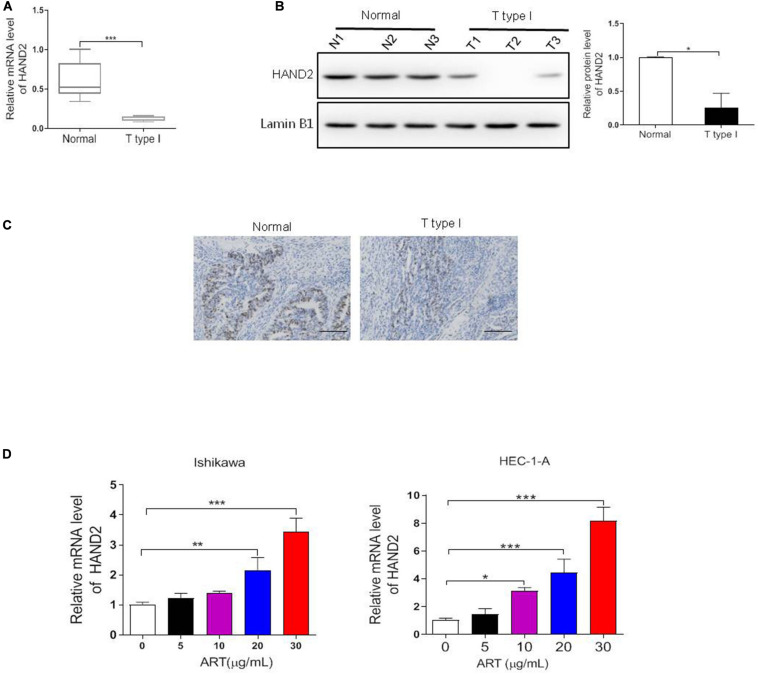
Heart and neural crest derivatives expressed 2 (HAND2) was downregulated in the ER-α-positive EC and regulated by ART treatment. **(A–C)** The expression level of ER-α in type I EC clinical samples and normal endometrial tissues were assessed by qRT-PCR, western blot and IHC assays. *p < 0.05, ***p < 0.001 vs. the normal group. Bar = 100 μm. **(D)** Ishikawa and HEC-1-A cells were treated with ART at different concentrations (0–30 μg/mL) for 4 days. The mRNA level of HAND2 was assessed by qRT-PCR analysis. The data were showed as the mean ± SD, *p < 0.05, **p < 0.01, ***p < 0.001 vs. the 0 μg/mL of ART-treated group.

Since HAND2 directly interacts with the ER-α and exerts a potential antitumor effect in EC (Fukuda et al., 2015), the effects of ART on HAND2 expression were then examined. In this study, we first determined the expression level of HAND2 in our clinical samples. As shown in Figures 4A,B, the mRNA and protein levels of HAND2 were both markedly downregulated in the type I EC (ER-α-positive) samples compared to the normal ones. Moreover, as shown in Figure 4C, the ER-α-positive patients presented a lower level of HAND2 than the normal tissue samples. To explore the potential effects of ART on HAND2 expression, we treated Ishikawa and HEC-1-A cells with 0, 5, 10, 20, 30 μg/mL of ART for 4 days and performed qRT-PCR analysis. The results showed that ART dose-dependently increased the expression level of HAND2 in these cells (Figure 4D).

### Effect of HAND2 on the Proliferation of ER-α-Positive EC Cells

**FIGURE 5:**
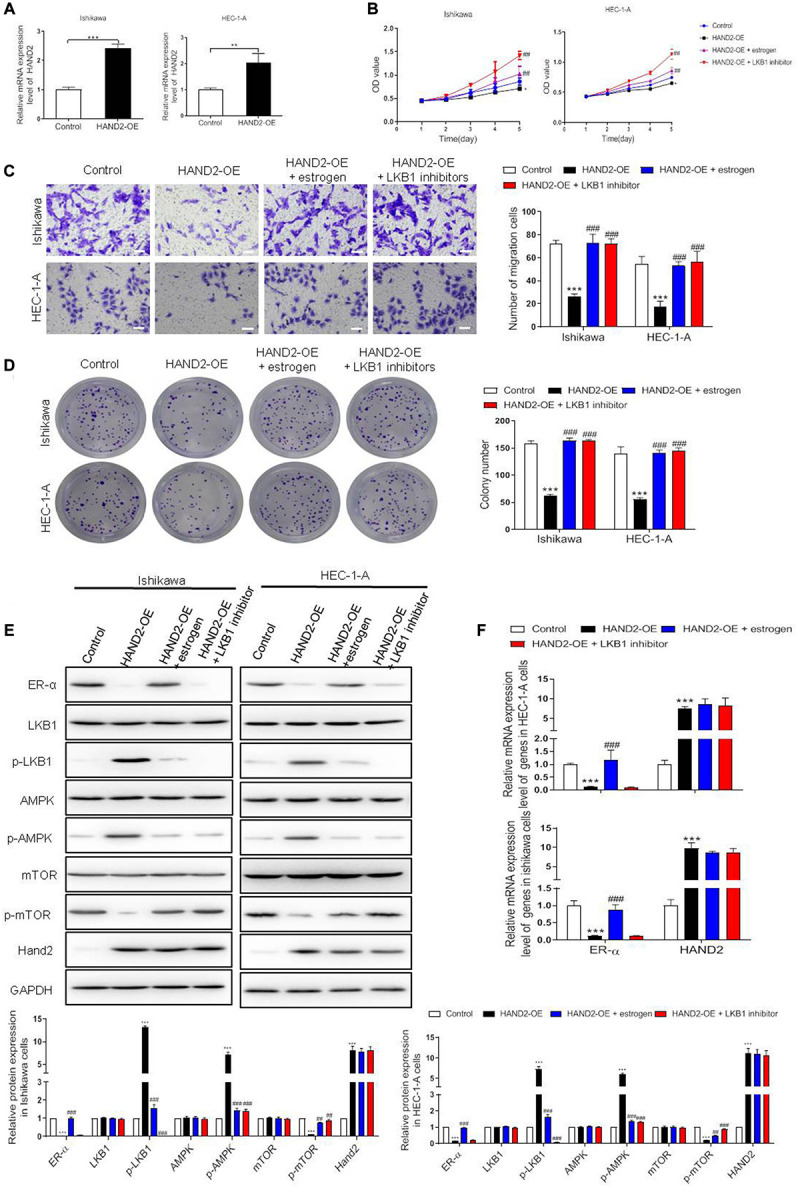
Effect of HAND2 on the proliferation of Ishikawa and HEC-1-A cells. **(A)** The efficiency of HAND2 overexpressed (HAND2-OE) vector transfection was assessed by qRT-PCR analysis. **(B)** These cells transfected with the Hand-OE vector and combining treatment with estrogen (10 nM) or LKB1 inhibitor (380 nM). Cell viability was assessed using CCK-8 assay. **(C,D)** The migration and colony formation assays were performed in these cells after different treatments. Bar = 20 μm. **(E)** Representative blot and analyses were performed for the protein levels of ER-α, LKB1, p-LKB1, AMPK, p-AMPK, mTOR, p-mTOR, and HAND2 in these cells. **(F)** The mRNA levels of ER-α and HAND2 were performed by qRT-PCR analysis. The data were showed as the mean ± SD, **p < 0.01, ***p < 0.001 vs. the control group; ##*p* < 0.01, ###*p* < 0.001 vs. the HAND2-OE group.

To determine the contribution of HAND2 on the proliferation of ER-α-positive EC cells, HAND2 was overexpressed by HAND2-OE vector transfection in Ishikawa and HEC-1-A cells. Overexpression of HAND2 led to an about two-folds increase in both cells compared with the empty vector-transfected cells (control, Figure 5A). As expected, HAND2-OE treatment significantly decreased the cell viability in these cells compared with the control group. When treated the HAND2 silenced cells with the estrogen, the cell viability was re-increased. Besides, the cells received LKB1 inhibitor (Pim1/AKK1-IN-1) treatment presented the highest viability level than other groups (Figure 5B). Furthermore, HAND2 overexpression also effectively downregulated cell migration or colony formation in these cells, and these effects were significantly reversed when combined treated with estrogen or LKB1 inhibitor (Figures 5C,D). Consistently, immunoblotting data indicated that upregulation of HAND2 inhibited the ER-α expression and decreased mTOR protein phosphorylation, while enhanced LKB1 and AMPK phosphorylation. Treatment with estrogen or LKB1 inhibitor abolished the activate effect of HAND2 on the LKB1/AMPK/mTOR pathway (Figure 5E). We also found that overexpressing of HAND2 resulted in a notable downregulation in the mRNA level of ER-α. However, neither estrogen nor LKB1 inhibitor could affect the HAND2 expression in these cells (Figure 5F). In addition, we also show that HAND2 knockdown promoted the proliferation and migration of ER-α-positive Ishikawa and HEC-1A cells. The silencing of HAND2 also upregulated the expression of ER-α at the mRNA and protein levels (Supplementary Figure 1). These findings suggested that HAND2 negatively regulated ER-α expression in estrogen-sensitive EC cells and thus led to the inactivation of downstream LKB1/AMPK/mTOR pathway.

### ART Inhibited EC Development in HAND2-Dependent Pathway

**FIGURE 6:**
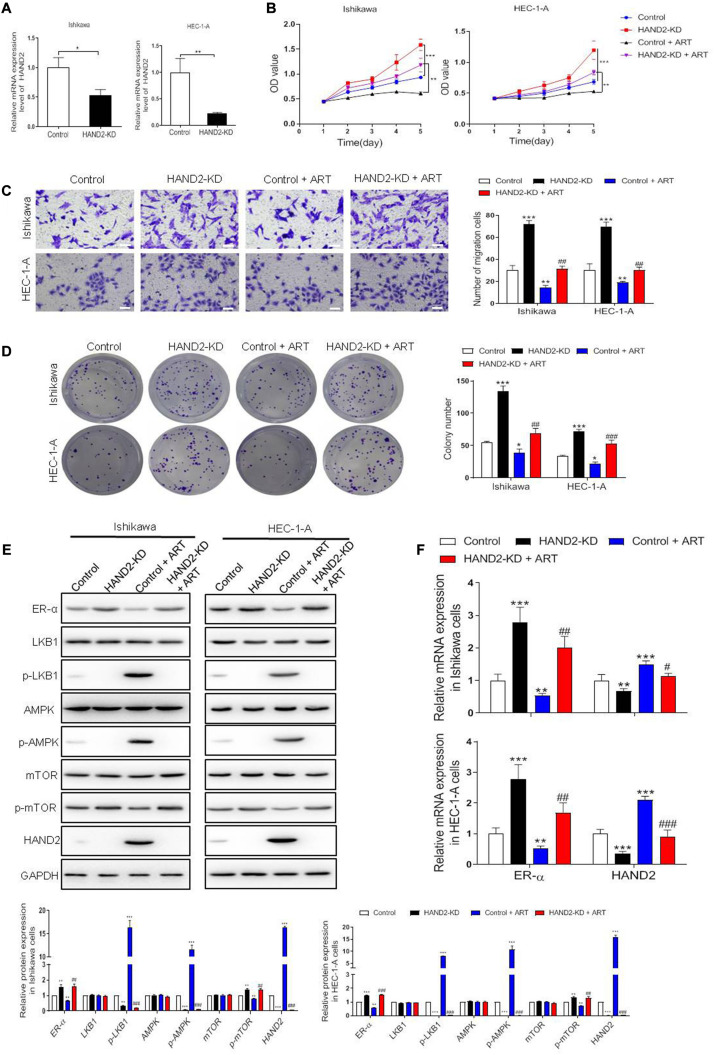
Artesunate (ART) inhibited the proliferation of Ishikawa and HEC-1-A cells via the HAND2 dependent pathway. **(A)** The efficiency of HAND2 downregulated (HAND2-KD) vector transfection was assessed by qRT-PCR analysis. **(B)** Cell viability was assessed in these cells transfected with the Hand-KD vector in the presence or absence of ART (30 μg/mL) treatment using CCK-8. **p < 0.01, ***p < 0.001. **(C,D)** The migration and colony formation assays were performed in these cells after different treatments. Bar = 20 μm. **(E)** Representative blot and analyses were performed for the protein levels of ER-α, LKB1, p-LKB1, AMPK, p-AMPK, mTOR, p-mTOR, and HAND2 in these cells. **(F)** The mRNA levels of ER-α and HAND2 were performed by qRT-PCR analysis. The data were showed as the mean ± SD, *p < 0.05, **p < 0.01, ***p < 0.001 vs. the control group; #*p* < 0.05, ##*p* < 0.01, ###*p* < 0.001 vs. the control + ART group.

Next, we silenced HAND2 expression in Ishikawa and HEC-1-A cells by transferring the HAND2-KD vector. The results showed that the silencing of HAND2 significantly increased cell viability, promoted cell migration in the ER-α-positive cells compared with the control group. Subsequently, exposure to ART suppressed the increase of proliferation and migration induced by HAND2 knockdown (Figures 6A–D). Consistently, western blot analysis demonstrated that the levels of proliferation-related proteins, LKB1 and AMPK downregulated while the level of mTOR upregulated in these HAND2 silenced cells, but these functions could be abolished by ART administration. In addition, we found knockdown of HAND2 upregulated ER-α expression at the mRNA and protein levels. However, compared with the control + ART group, the silencing of HAND2 in the cells significantly reversed the suppression effect of ART on ER-α (Figures 6E,F).

**FIGURE 7:**
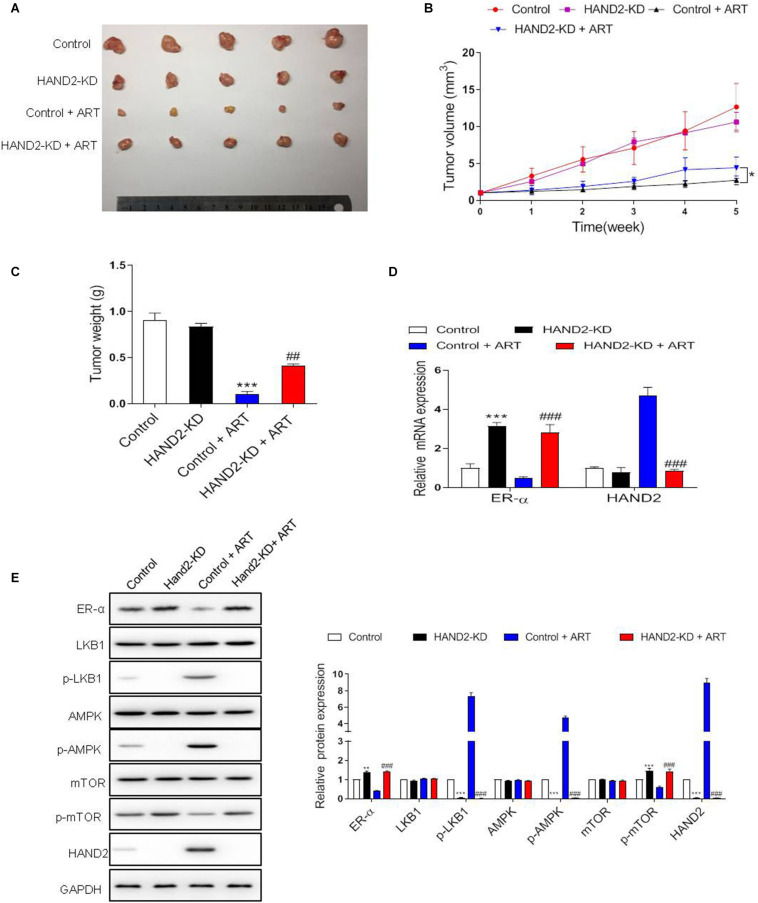
Artesunate (ART) inhibited tumor proliferation via the HAND2 dependent pathway. After transfected with HAND2-KD, the Ishikawa cells injected subcutaneously into mice, followed by ART treatment (30 mg/kg/d, in 200 μL PBS). **(A)** Typical photos of tumors from control, HAND2-KD, control + ART, and HAND2-KD + ART groups 5 weeks after injection. **(B,C)** The tumor volume and weight of these groups. *p < 0.05. **(D)** The mRNA levels of ER-α and HAND2 were performed by qRT-PCR analysis. **(E)** Representative blots and analyses were performed for the expressions of ER-α, LKB1, p-LKB1, AMPK, p-AMPK, mTOR, p-mTOR, and HAND2 in the tumor tissues of mice. The data were showed as the mean ± SD, **p < 0.01, ***p < 0.001 vs. the control group; ##*p* < 0.01, ###*p* < 0.001 vs. the control + ART group.

To determine the effect of HAND2 on tumor growth *in vivo*, we generated the EC model through subcutaneous injection of Ishikawa cells. At 5 weeks after injection, the tumor volume and weight in HAND2-KD transfected cells transplanted mice were similar to that in the control group. In contrast, the tumor size and weight were dramatically reduced by ART treatment than the control group, while the effect was significantly abolished when knockdown HAND2 expression (Figures 7A–C). These findings indicated that HAND2 was required for the antitumor effect of ART. Besides, western blot results showed that the expression of ER-α, p-mTOR was decreased while the levels of HAND2, p-LKB1, and p-AMPK were both increased in the control + ART group. However, the silencing of HAND2 reversed the antitumor effect of ART with re-upregulated ER-α expression and inhibited the phosphorylation of key markers of LKB1/AMPK/mTOR pathway (Figures 7D,E).

## Discussion

A high level of ER-α expression has been detected in the endometrioid type of EC, particularly in the early stage. Targeting the ER-α may represent an effective therapy option for EEC. In this study, we demonstrated that aberrant expression of ER-α was essential for tumor proliferation and was associated with a good prognosis in the clinic. Besides, patients with ectopic ER-α expression presented the activation of genes in the LKB1/AMPK pathway and suppression of mTOR phosphorylation. Recent studies have suggested that the LKB1/AMPK pathway acted as the suppressor for multiple types of cancer. Considering that LKB1 is a target gene of ER-α, the expression of such genes might rely on the ER-α status in EC. Here, we changed the expression level of ER-α using fulvestrant or estrogen in ER-α-positive EC cells, Ishikawa and HEC-1-A cells. The results showed that estrogen upregulated ER-α expression at both mRNA and protein levels along with the inhibition of the LKB1/AMPK pathway. In contrast, fulvestrant suppressed the expression of ER-α and activated this pathway. Taken together, our findings supported that ER-α served as a tumor-promoting factor for EC via inhibiting the LKB1/AMPK pathway.

It was reported that ERα-engaged infiltrating macrophages initiated chronic inflammation and promoted the aggressive progression of endometrial cancer cells (Jing X. et al., 2019). In endometriosis rats, anti-inflammatory agent could attenuate disease progression (Di Paola et al., 2016). ART showed remarkable anti-inflammatory effects in various disease (Gugliandolo et al., 2018, 2020). Recent studies have demonstrated the antitumor effect of artesunate in several cancer types. One study revealed that ART inhibited the cell proliferation of MCF-7 and MDA-MB-231, two cell types of breast cancer, but ER-positive MCF-7 cells were more sensitive to ART treatment than the ER-negative MDA-MB-231 cells (Tran et al., 2016). However, the activity of ART in EC remains unknown. In the present study, we showed that ART could induce cell cycle arrest, apoptosis, and inhibit migration of ER-positive endometrial cancer cells dose-dependently. Moreover, using the *in vivo* model of mice, ART suppressed the proliferation of xenograft tumors by intramuscular administration. Notably, ART inhibited ER-α expression and stimulated downstream LKB1/AMPK pathway, which was reversed by combined treatment with the agonist of ER-α, estrogen. In addition, we also found that ART treatment reduced the cell viability of the ER-α negative EC cells. However, it was unresponsive to this pathway upon ART treatment (Supplementary Figure 2). These findings suggested that artesunate could activate the LKB1/AMPK/mTOR pathway to trigger apoptotic responses in the ER-α dependent manner.

Heart and neural crest derivatives expressed 2 is a transcriptional factor involved in the maintaining of endometrial function (Kato et al., 2019). The methylation and silence of HAND2 contributed to the high risk of EC (Liew et al., 2019; Multinu et al., 2020). Based on the immunoprecipitation and pull-down assay, one study also pointed out that HAND2 directly interacted with the amino-terminus of ER-α, resulted in inhibition of the transcriptional expression of it (Fukuda et al., 2015). Therefore, HAND2 was considered as a critical factor in EC progression. Notably, our previous and present studies revealed that HAND2 was less expressed in the ER-α positive endometrial tumor tissues compared with the normal endometrial tissues (Yin et al., 2017). Upon the ART administration, the expression level of HAND2 in Ishikawa and HEC-1-A cells were both increased dose-dependently. We thus hypothesized that whether ART regulated the expression of ER-α in the HAND2-dependent manner. In the ER-α-positive cells, we confirmed that HAND2 negatively regulated the expression of ER-α. In addition, overexpression of HAND2 could significantly enhance the antitumor effect of ART, while it was reversed by estrogen or LKB1 inhibitor treatment. However, the silencing of HAND2 in the ER-α-positive cells increased the cell growth even in the presence of ART treatment. Activation of ER-α by estrogen or activation of LKB1 by metformin both abolished the promoting effect induced by HAND2. Besides, the *in vivo* study also indicated that the downregulation of HAND2 obviously inhibited the antitumor effect of ART via inactivating of ER-α moderated LKB1/AMPK pathway.

Our work collectively elucidates the antitumor effect of ART on ER-α-positive EC via inducing cell apoptosis and cell cycle arrest and reducing cell migration. These findings also revealed that ART regulated the expression of ER-α in the HAND2-dependent manner.

## Data Availability Statement

The original contributions presented in the study are included in the article/Supplementary Material, further inquiries can be directed to the corresponding author/s.

## Ethics Statement

The studies involving human participants were reviewed and approved by the Ethics Committee of Clinical Medical College of Yangzhou University. The patients/participants provided their written informed consent to participate in this study. The animal study was reviewed and approved by Clinical Medical College of Yangzhou University.

## Author Contributions

All authors contributed to this review with conception and design, literature review, drafting and critical revision, editing, and approval of the final version.

## Conflict of Interest

The authors declare that the research was conducted in the absence of any commercial or financial relationships that could be construed as a potential conflict of interest.

## Supplementary Material

Supplementary Figure 1 Effect of HAND2 on the proliferation of Ishikawa and HEC-1-A cells. **(A)** The efficiency of HAND2 downregulated vector transfection was assessed by qRT-PCR analysis. **(B)** Cell viability was assessed in these cells transfected with Hand-KD vector and combining treatment with fulvestrant (10 nM) or LKB1 agonist (metformin, 5 mM in PBS, MedChemExpress, United States) using CCK-8 assay. **(C,D)** The migration and colony formation assays were performed in these cells after different treatments. Bar = 20 μm. **(E)** Representative blot and analyses were performed for the protein levels of ER-α, LKB1, p-LKB1, AMPK, p-AMPK, mTOR, p-mTOR, and HAND2 in these cells. **(F)** The mRNA levels of ER-α and HAND2 were performed by qRT-PCR analysis. The data were showed as the mean ± SD, ^∗∗^*p* < 0.01, ^∗∗∗^*p* < 0.001 vs. the control group.

Supplementary Figure 2 ART inhibited the proliferation of ER-α-positive cells via the HAND2 dependent pathway. The ER-α-positive Ishikawa cells were treated with (A) 30 μg/mL of ART or (B) control for 4 days. The ER-α-positive HEC-1-A cells were treated with (C) 30 μg/mL of ART or (D) control for 4 days. The ER-α-negative JEC cells were treated with (E) 30 μg/mL of ART or (F) control for 4 days. The western blot and qRT-PCR analysis were performed for the expression of ER-α, p-LKB1, p-AMPK, p-mTOR, and HAND2 in these cells after different treatments.

## Abbreviations

EC: endometrial carcinoma
ART: artesunate
ER- α: estrogen receptor- α
EC: endometrial cancer
ER: estrogen receptor
AMPK: AMP-activated protein kinase
LKB1: liver kinase B 1.
