# Supplementary figures and images for: Artesunate Suppresses the Proliferation and Development of Estrogen Receptor-α-Positive Endometrial Cancer in HAND2-Dependent Pathway
Source: Front Cell Dev Biol. 2021 Jan 12;8:606969. doi: 10.3389/fcell.2020.606969 (PMC7835542; doi:10.3389/fcell.2020.606969)

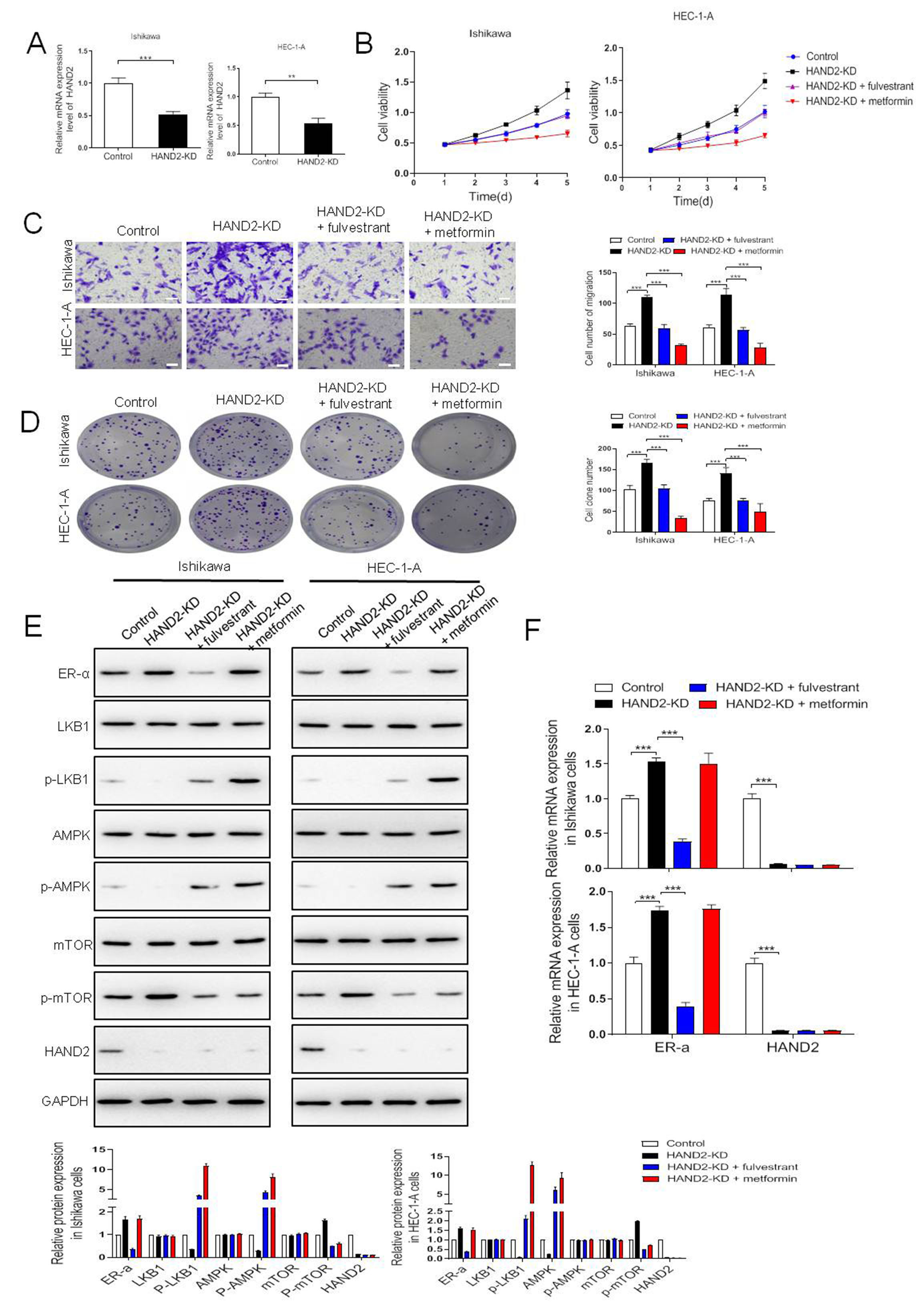

Supplement: Supplementary Figure 1 — Effect of HAND2 on the proliferation of Ishikawa and HEC-1-A cells. (A) The efficiency of HAND2 downregulated vector transfection was assessed by qRT-PCR analysis. (B) Cell viability was assessed in these cells transfected with Hand-KD vector and combining treatment with fulvestrant (10 nM) or LKB1 agonist (metformin, 5 mM in PBS, MedChemExpress, United States) using CCK-8 assay. (C,D) The migration and colony formation assays were performed in these cells after different treatments. Bar = 20 μm. (E) Representative blot and analyses were performed for the protein levels of ER-α, LKB1, p-LKB1, AMPK, p-AMPK, mTOR, p-mTOR, and HAND2 in these cells. (F) The mRNA levels of ER-α and HAND2 were performed by qRT-PCR analysis. The data were showed as the mean ± SD, ∗∗p < 0.01, ∗∗∗p < 0.001 vs. the control group. [file Image_1.tif]

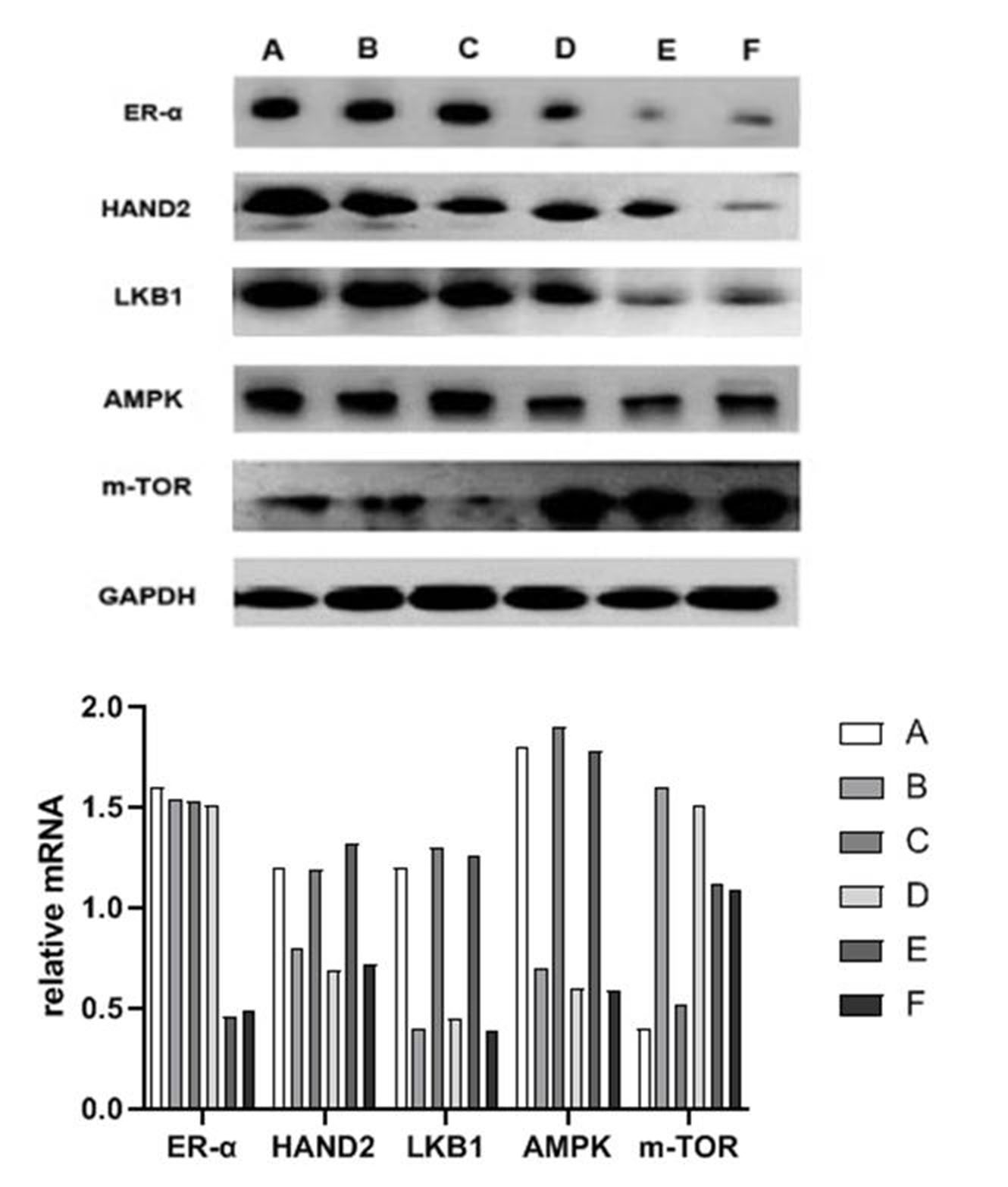

Supplement: Supplementary Figure 2 — ART inhibited the proliferation of ER-α-positive cells via the HAND2 dependent pathway. The ER-α-positive Ishikawa cells were treated with (A) 30 μg/mL of ART or (B) control for 4 days. The ER-α-positive HEC-1-A cells were treated with (C) 30 μg/mL of ART or (D) control for 4 days. The ER-α-negative JEC cells were treated with (E) 30 μg/mL of ART or (F) control for 4 days. The western blot and qRT-PCR analysis were performed for the expression of ER-α, p-LKB1, p-AMPK, p-mTOR, and HAND2 in these cells after different treatments. [file Image_2.tif]
